# Stimulating Influenza Vaccination via Prosocial Motives

**DOI:** 10.1371/journal.pone.0159780

**Published:** 2016-07-26

**Authors:** Meng Li, Eric G. Taylor, Katherine E. Atkins, Gretchen B. Chapman, Alison P. Galvani

**Affiliations:** 1 Department of Health and Behavioral Sciences, University of Colorado Denver, Denver, Colorado, United States of America; 2 Center for Infectious Disease Modeling and Analysis, Yale School of Public Health, New Haven, Connecticut, United States of America; 3 Department of Infectious Disease Epidemiology, London School of Tropical Medicine and Hygiene, London, United Kingdom; 4 Department of Psychology, Rutgers University, New Brunswick, New Jersey, United States of America; Chang Gung Memorial Hospital, TAIWAN

## Abstract

**Objective:**

Americans do not vaccinate nearly enough against Influenza (flu) infection, despite severe health and economic burden of influenza. Younger people are disproportionately responsible for transmission, but do not suffer severely from the flu. Thus, to achieve herd immunity, prosocial motivation needs to be a partial driver of vaccination decisions. Past research has not established the causal role of prosociality in flu vaccination, and the current research evaluates such causal relationship by experimentally eliciting prosociality through messages about flu victims.

**Methods:**

In an experimental study, we described potential flu victims who would suffer from the decision of others to not vaccinate to 3952 Internet participants across eight countries. We measured sympathy, general prosociality, and vaccination intentions. The study included two identifiable victim conditions (one with an elderly victim and another with a young victim), an unidentified victim condition, and a no message condition.

**Results:**

We found that any of the three messages increased flu vaccination intentions. Moreover, this effect was mediated by enhanced prosocial motives, and was stronger among people who were historical non-vaccinators. In addition, younger victim elicited greater sympathy, and describing identifiable victims increased general sympathy and prosocial motives.

**Conclusions:**

These findings provide direct experimental evidence on the causal role of prosocial motives in flu vaccination, by showing that people can be prompted to vaccinate for the sake of benefiting others.

## Introduction

Imagine Walter, who was 76 years old and was not able to get the flu vaccine last year due to mobility issues. Walter relied on other people to get vaccinated to reduce the chance that they spread the virus to him. Last year, many people neglected to get the Influenza (flu) vaccine, and Walter became infected. As a result of his infection, he acquired pneumonia. He was in a critical condition for several weeks before passing away.

Flu infection leads to about 200,000 hospitalizations [1], 610,660 life-years lost, and $87.1 billion financial cost [2] in the U.S. each year. Although flu vaccination is the most effective way to prevent the flu, Americans do not vaccinate even close to the 70% rate set by the 2020 Healthy People initiative [3]. The story about Walter highlights the societal effect of vaccination: Flu vaccination reduces the risk of onward transmission to others in addition to reducing the risk of infection for oneself, a phenomenon known as herd immunity [4]. This “herd immunity” entails that flu vaccination is a prosocial behavior.

Prosocial behavior is defined as behavior that benefits others, whether or not it involves an overall cost to self [5]. Prosocial behavior has received extensive attention in psychological research, as it includes a variety of important social behaviors such as helping, sharing, cooperation, and charitable donation etc [6, 7]. Flu vaccination benefits other people through herd immunity. Thus, we use the term “prosociality” in this paper to discuss the motivation in flu vaccination that is intended to help others.

Prosocial motivation is especially critical in flu vaccination because flu transmission and morbidity is asymmetric: Young people are responsible for the majority of transmission [8], but in a typical year, 90% of flu-related deaths happen to those above 65 years of age [9]. Thus, the most efficient way to reduce the societal disease burden of flu is for the majority of young people below 65 years old to vaccinate and bear the cost of doing so (fee, inconvenience, and perceived side effects), even though they experience less personal benefit from vaccination[10]. That is, prosocial motivations, or the concern to benefit others, need to affect flu vaccination to achieve the optimal vaccine coverage[10]. In this paper, we ask whether prosocial motives do in fact lead people to vaccinate against the flu.

### Past research on prosocial motivation and vaccination

Existing research suggests that prosocial motives do play a role in vaccination decisions. For instance, in scenarios about hypothetical vaccines, study participants were more likely to receive the vaccine when they learned that their vaccination can benefit more people, [11, 12], when the social benefit of vaccination was emphasized and cost of vaccination was low [13], or when they were incentivized to care about others in a simulated flu game [14]. Shim et al. [15] also found a positive association between concerns for spreading the flu to others and the decision to vaccinate, even after controlling for self-interested concern about contracting the flu. These findings are limited, however, either by the hypothetical nature of the vaccine in the scenarios, or by the correlational design of the study.

In parallel, research on parental vaccination decisions also indicates that “there may be a general willingness to immunize children for the benefit of others” [16], but the evidence comes largely from interviews or questionnaires directly asking parents their reasons to vaccinate. In contrast, a recent experimental study found that emphasizing societal benefit did not increase parents’ intentions to vaccinate their child with the MMR vaccine without mentioning benefits to the child [17]. Thus, the existing evidence does not provide conclusive support for the causal impact of prosocial motives on vaccination decisions.

### The current study

The goal of the current study was to provide a stringent test of whether prosocial motives have a direct causal impact on vaccination decisions in the context of flu vaccination. We aimed to experimentally elicit prosocial motivation using messages, and in turn, to promote flu vaccination. How can we effectively activate prosocial motivation in flu vaccination? Past research on the reason for prosocial behavior has demonstrated that greater empathy [18] and a heightened sense of personal responsibility [19] towards the victim are powerful motivating factors in prosocial behavior. We drew on this research to create several messages: To increase empathy and personal responsibility, we described the negative consequences that flu victims might suffer, and emphasized the personal responsibility of those who failed to vaccinate for such consequences.

We used an experimental design to clearly delineate the causal role of prosociality. The experiment included a control condition where no message about flu vaccination was presented, and three experimental conditions were different versions of prosocial messages were presented. As outcome variables, we measured participants’ prosocial motivation and intentions to receive a flu vaccine. Because participants were randomly assigned to different conditions, all other factors that can contribute to flu vaccination, such as preexisting attitudes about the vaccine, risk perceptions, perceived social norm etc. [20, 21], are equated across conditions, leaving the experimental manipulation the unambiguous cause of any potential difference in the outcome variables. To our knowledge, this is the first experimental study directly testing the causal role of prosocial motivation in flu vaccination.

Among the three versions of the prosocial messages, we also tested whether description of identifiable victims may be more effective in eliciting prosocial motives than description of unidentified victims. This research question was informed by past research on charitable giving, which shows that describing a victim with identifying details (e.g., name, age, picture, short story) elicits a powerful emotional response [22], and generates more charitable giving compared to similar descriptions of unidentified victims [23–26]. We designed two messages that described a flu victim (one using a young victim, and another using an old victim) with name, age, picture, and a short story, and compared them with another message that described flu victims in general without these identifying details (see Methods).

As part of a larger survey about international views on vaccination [27], this study was conducted among an Internet panel across 8 countries.

## Materials and Methods

### Data collection

We requested 500 completed Internet survey responses per country from 8 countries through the commercial survey company Research Now (RN). RN recruited 4023 participants before the start of each country’s flu season from China (N = 499), France (N = 497), Japan (N = 501), United Kingdom (N = 496) and United States (N = 473) during September 13–23, 2013, Israel (N = 500) during October 1–5, 2013 (to avoid a major Israeli holiday), and Brazil (N = 490) and South Africa (N = 496) during March 10–18, 2013. Participants were recruited either from the RN or affiliate panelist pools. We excluded 63 respondents whose IP address conflicted with their country of residence in the RN database, 6 respondents who were younger than 18 years old, and 2 responses from participants who took the survey a second time, yielding 3952 final responses. The response rates in each country ranged from 2% (China) to 20% (Japan), with a median of 10.5%.

We set quotas within each country where feasible to achieve a representative sample across age and gender (See Table 1). We aimed to achieve an equal balance of male and female participants for greater representativeness of the actual population. All countries except Israel and South Africa (which had limited Internet participants) had a gender quota of 50% male and 50% female. Because of younger people tend to be over represented among Internet users, we also set age quotas to ensure a decent representation of older participants. For France, Japan, United Kingdom and United States, we specified 20% participants should be 50 years or older; in Brazil we adjusted this quota to 10%, and in China we specified that 30% of participants should be 35 years or older. We set no age quota in Israel due to limitations in the size of the participant pool available.

**Table 1:** Participant Age and Gender Distribution by Country.

Country	*n*	*M*	*SD*	*Med*	*Min*	*Max*	% 35 years or older	% 50 years or older	% of female
Brazil	490	35.44	11.13	35	18	77	50.4	9.8	51
China	499	33.13	9.81	31	19	89	29.7	6.8	50
France	497	40.02	12.97	38	18	77	59.6	20.1	50
Israel	500	38.77	15.01	33	18	98	46.6	28.8	58
Japan	501	39.90	13.53	37	18	100	57.9	20.0	50
SA	496	37.60	12.18	35	18	74	52.8	17.5	60
UK	496	40.66	12.80	40	18	80	63.5	20.2	50
US	473	40.49	12.53	38	19	82	60.5	20.1	49

The study was part of a larger survey about seasonal flu and flu vaccination [28], which included questions about participants’ flu vaccination history, contact patterns, how people influence each others’ vaccination decisions, as well as hypothetical scenarios about flu vaccination. All study materials were translated into the official language of the respective countries (Portuguese, Chinese, French, Hebrew, Japanese, British English, and American English) and back translated to ensure accuracy. We initiated the survey in the primary language of the participant’s location, but participants could also select any of the 7 languages above.

We obtained IRB approval at Rutgers University (IRB protocol 12–782), Yale University (IRB protocol 120710537) and University of Colorado Denver (IRB protocol 12–1212). All participants read the consent form (translated into appropriate language) before participating in the study, and assented to the study by clicking a button to proceed to the next page of the survey. The consent procedure was approved by the IRB, and recorded as part of the dataset.

### Questionnaire

The current study was positioned at the end of the larger survey. Participants were randomly assigned to one of four conditions: They either read a message about flu vaccination that involved an identified young victim, an identified elderly victim, a general message about unidentifiable flu victims, or no message. We included the elderly victim condition because they are the usual victims of severe flu-related illnesses, and included the young victim condition because children under 6-months of age are not eligible for the flu vaccine [9], and thus, rely solely on the vaccination of people around them for protection. Another reason for including the young victim condition was to see whether young victims could elicit greater prosocial motives, as past research has shown that people value lives of younger people more than older people [29–31].

In the two identifiable victim conditions (young victim or elderly victim), participants were informed: “The persons described in the following scenario are fictional but their stories are based on true events.” The elderly victim scenario described a 76-year old man who contracted flu and eventually died last year because others had neglected to vaccinate; the young victim scenario described a 5-month old boy. Both messages included name and photo of a smiling victim, varied by country, and age of victim [24–26]. The unidentified victim message described how neglecting to vaccinate can cause the flu to spread to others, who might die of complications, and included an image of a smiling a doctor to equalize the three message conditions. In the no message condition, no such scenarios were presented. We used images of white victims in European and American countries (France, Israel, UK, US, and Brazil), images of Asian victims in China and Japan, and images of black victims in South Africa. All images portrayed male victims, to avoid the cofounding effect of victim gender across countries. See S1 Supporting Information for original messages. The images are not presented in the paper for confidentiality reasons.

The name of the younger and older character as well as the appearance of the person in the images were adapted to represent the majority population in the European/American countries (France, Israel, UK, and US), Asian countries (China and Japan), and African Country and (South Africa), respectively. Participants in the three message conditions also answered two comprehension questions about the message, and re-read the message again if they answered either question incorrectly.

We measured three variables: sympathy, general prosociality, and vaccination intentions (see S1 Supporting Information). We asked participants how much sympathy they felt for the flu victim who died (elderly & young victim conditions) or victims who may have died (general condition). Prosocial motive was measured as the likelihood that the participant would donate money to the Against Malaria Foundation. (We also asked how much they would be willing to donate, but did not analyze the amount due to the complication of different currencies and wages across countries). To determine vaccination intention, we asked whether participants had already received the flu vaccine this year, and if not, how likely they were to get the flu vaccine this coming flu season. We reminded participants to be objective and consider not only the potential benefits of vaccination, but also any reservations they may have and practicalities involved, such as making the appointment and going to the clinic. Sympathy was measured on a seven-point scale from 1 “very little” to 7 “very much”, and likelihood to donate and vaccinate were both measured on a scale from 0% to 100%, in 10% increments. We measured these variables in all four conditions, except sympathy, which we only asked in the three message conditions, where victims were involved. We randomly assigned the order of the sympathy and general prosociality measures, and asked likelihood to vaccinate question last.

### Summary of analysis approach

To analyze the effect of message conditions on sympathy, prosociality, and vaccination intentions, we used Analysis of Variance (ANOVA) to test the overall difference of each outcome variable across 4 conditions (except for sympathy, only 3 conditions were available), as well as focused contrasts to test whether the three conditions containing message differed from the no-message condition, whether the two identified message conditions differed from the general message condition, and whether the young victim condition differed from the old victim condition. Where homogeneity of variance was not met, Welch test was reported. Similar analyses accounting for country, age, and gender as covariance were conducted using Analysis of Covariance (ANOCOVA), with details described in S2 Supporting Information. We also performed meditational analyses with sympathy as a potential mediator for the effect of identifiable victim on prosociality, and with prosociality as a potential mediator for the effect of message (vs. no message) on vaccination intentions, both using the PROCESS macro developed by Hayes [32], which is based on bootstrapping estimation of the indirect effect.

In the analyses on vaccination intentions, we also performed analyses among all participants, as well as among participants who did versus did not receive flu vaccine in the prior year separately.

## Results

### Sympathy

All participants who read one of the three messages (N = 2968) reported sympathy towards the victim(s) of flu. A one-way ANOVA showed a significant overall effect of condition, Welch’s test (accounting for heterogeneity of variance) = 58.64, *p* < .001. Focused contrasts showed that the young victim message (M = 6.05, 95% CI [5.96, 6.14]) elicited greater sympathy than the old victim message (M = 5.56, 95% CI [5.46, 5.65]), *t* (1979.32) = 7.34, Cohen’s *D* = 0.33, *r* = .16, *p* < .001, and these two identifiable victim conditions pooled elicited greater sympathy than the general information condition (M = 5.32, 95% CI [5.22, 5.42]), *t* (1814.51) = 7.80, Cohen’s *D* = 0.37, *r* = .18, *p* < .001.

### Prosocial motives

**Fig 1:**
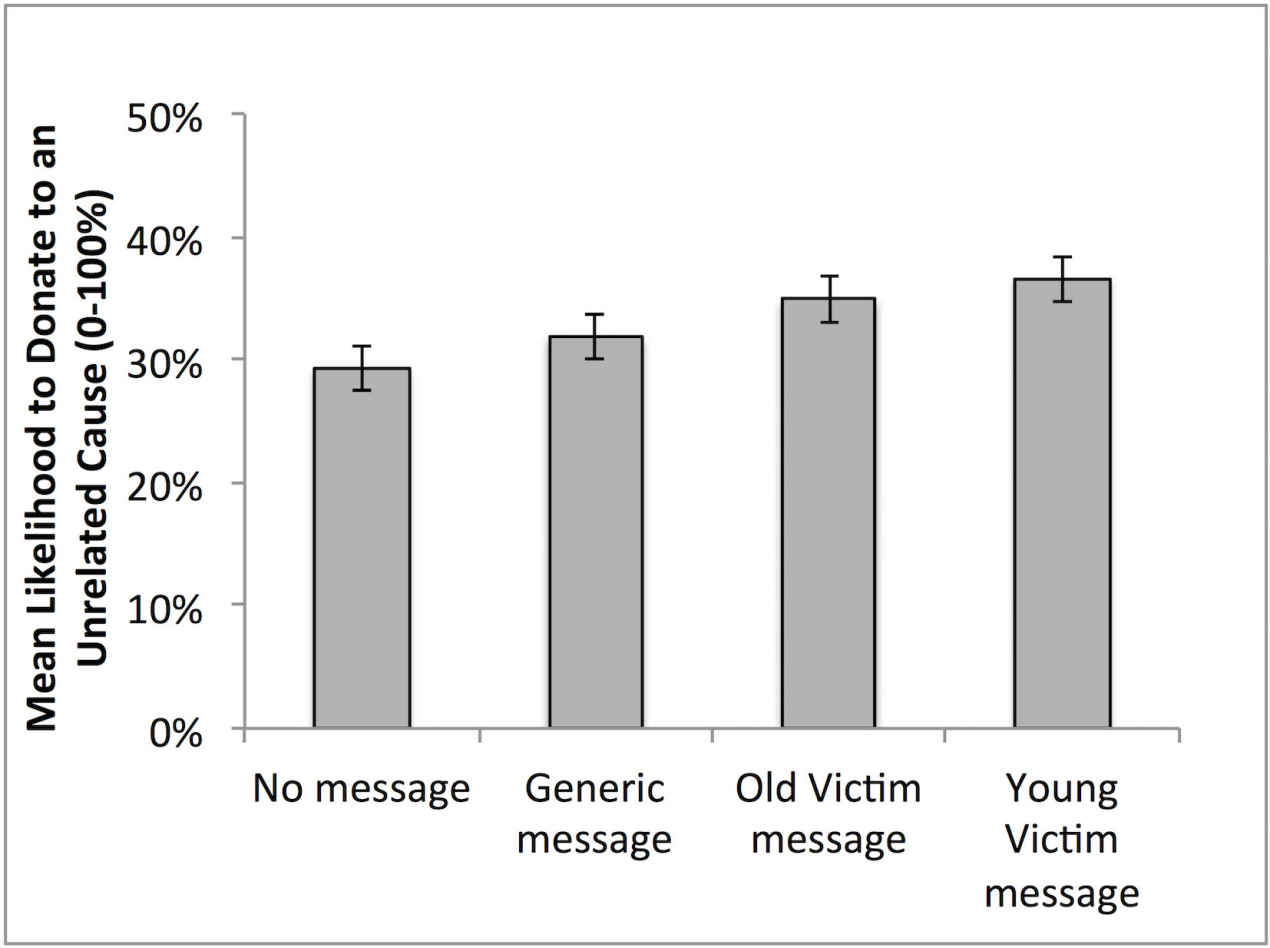
Mean Prosocial Motives as Measured in Rated Likelihood to Donate to an Unrelated Cause. Error Bars: ± 2SEs (95% CI).

Among all 3952 valid participants, a one-way ANOVA revealed a significant effect of message condition on prosocial motivation, as measured in likelihood to donate to an irrelevant cause, Welch’s test = 12.82, *p* < .001 (Fig 1). The three message conditions pooled elicited greater likelihood to donate compared to the no-message condition, *t* (1774.57) = 5.02, Cohen’s *D* = 0.24, *r* = .12, *p* < .001; similarly, the two identifiable victim conditions led to greater donation intentions compared to the general message condition, *t* (1966.53) = 3.41, Cohen’s *D* = 0.15, *r* = .08, *p* = .001. Young and old victim conditions led to similar likelihood to donate, *p* > .20.

**Fig 2:**
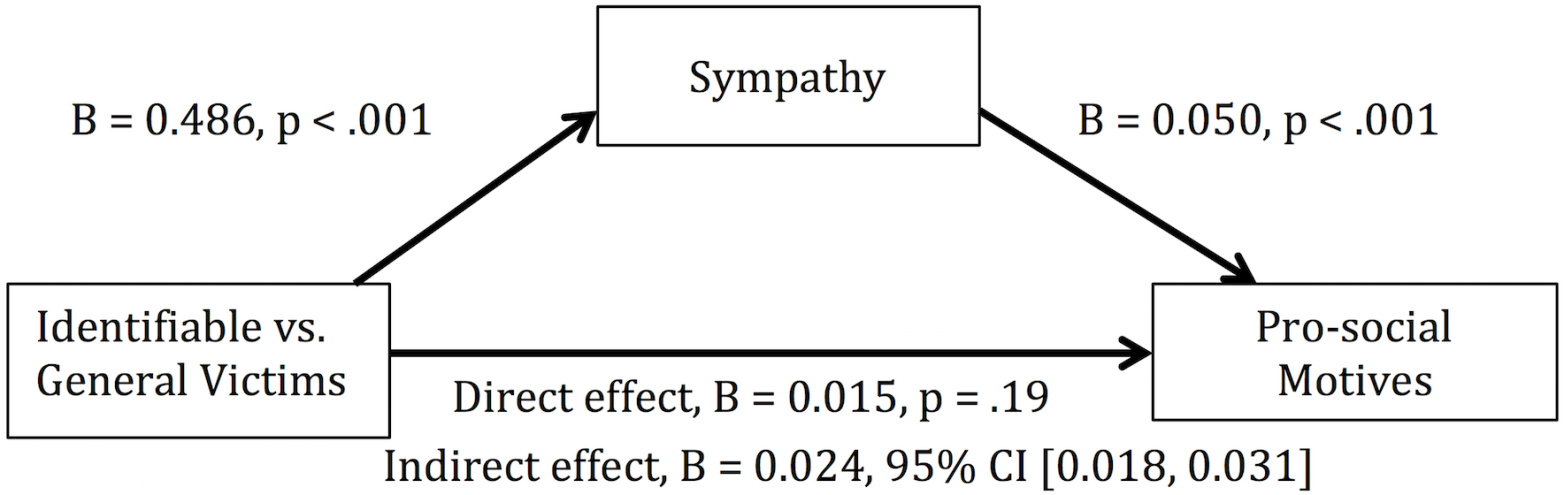
Mediational Model for the effect of identifiable victim on prosocial motives, mediated by sympathy. This mediation was based on the bootstrapping method for calculating indirect effect. Analysis was performed in SPSS using the PROCESS macro developed by Hayes [32], with 5000 resampling to compute the Bias Corrected boot strapped 95% Confidence Intervals (BCa CI). Total effect of identifiable victim on prosocial motive was significant, B = 0.039, BCa 95% CI [0.017, 0.061], p = .007.

As we expect sympathy to be a motivator of prosociality, we assessed the mediating role of sympathy in the effect of message on donation intentions. Given that sympathy was only assessed in the three message conditions, we were specifically interested in the role of sympathy in the elevated donation intentions among the identifiable victim messages compared to the general message. We conducted a mediation analysis using the contrast between the identifiable victim conditions and the unidentifiable victim condition as the predictor, donation intentions as the outcome variable, and sympathy as the mediator. We used the PROCESS Macro developed for SPSS by Hayes [32] to estimate the mediation effect, with a bias-corrected bootstrapping method and 5000 resampling (Fig 2). Sympathy significantly mediated the impact of the identifiable victim on prosociality, indirect effect B = 0.024, 95% CI [0.018, 0.031], which represents a small effect, *κ*^2^ = .039, 95% CI [0.029, 0.050]. This means that compared to the general message, the identifiable victim messages led to a 2.4% (0.024) absolute increase (on a percentage likelihood scale) in prosociality in response to identifiable victim messages because of heightened sympathy. Thus confirming the role of sympathy in prosocial messaging.

### Vaccination intentions

We analyzed vaccination intentions for 3518 participants who reported not to have received a flu vaccine for the upcoming season, and thus answered the vaccination intention question.

In a one-way ANOVA, vaccination intentions differed across the four conditions, F (3, 3514) = 5.40, partial *η*^*2*^ = .005. Focused contrast showed that the three message conditions produced significantly higher intentions to vaccinate than the no-message condition, *M*_*Message*_ = 44%, 95% CI [42%, 45%] vs. *M*_*No-message*_ = 38%, 95% CI [36%, 41%], *t* (3514) = 3.98, Cohen’s *D* = 0.13, *r* = .07, *p* < .01. This 6-percentage point absolute increase represents a 15% relative increase in the reported desire to receive a vaccine. Neither the contrast between the identifiable victim conditions and general information condition, nor the contrast between young and old victim conditions was significant (*p* > .57 for both).

**Fig 3:**
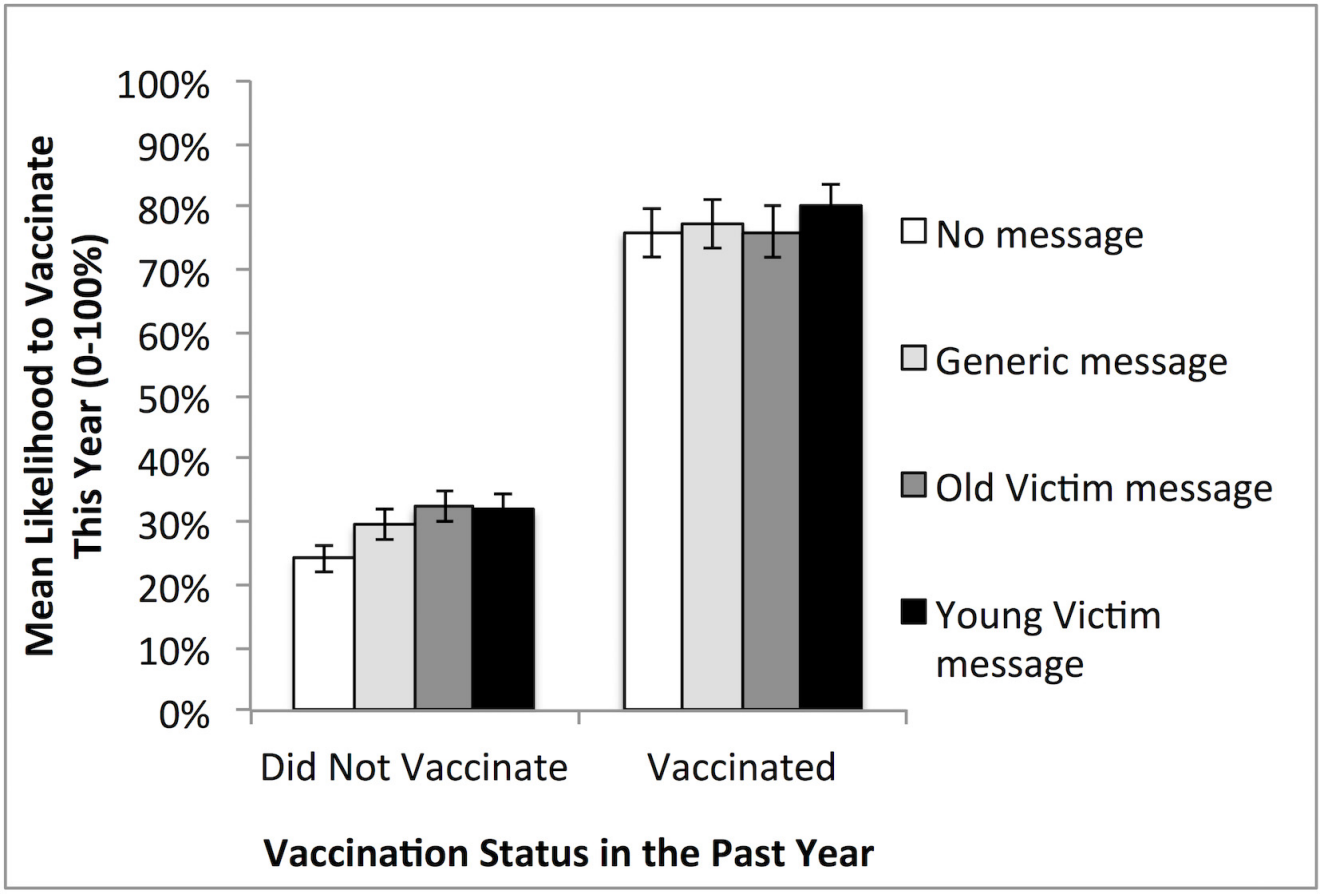
Mean Vaccination Intentions across Condition and Past Year Vaccination Status. Error Bars: ± 2SEs (95% CI).

Our data also confirmed a previous finding that one major predictor for flu vaccination of a given individual is prior vaccination behavior [20]: As shown in Fig 3, participants who had vaccinated in the prior year (n = 909) indicated a 77% likelihood to vaccinate this year on average, 95% CI [75%, 79%], with close to half of these participants (n = 418) indicating a 100% likelihood; conversely, participants who had not vaccinated in the prior year (n = 2530) indicated a much lower mean likelihood to vaccinate: 29%, 95% CI [28%, 31%], and only 4% (n = 93) of these participants indicated a 100% likelihood. Thus, the latter group is in particular need of interventions to encourage their vaccination.

We tested the effect of message for these two groups separately. Among those who had vaccinated in the previous year, vaccination intention did not vary significantly across conditions, either in omnibus test or contrast tests. This group of people was uniformly highly likely to vaccinate this year. But for participants who had not vaccinated in the previous year, the overall effect of message condition was significant, Welch’s test = 11.10, p < .001. Among these historical non-vaccinators, the contrast between the message conditions and the no-message was significant, *M*_*Message*_ = 31%, 95% CI [30%, 33%] vs. *M*_*No-message*_ = 24%, 95% CI [22%, 26%], *t* (1202.59) = 5.47, Cohen’s *D* = 0.32, *r* = .16, *p* < .01. Here, the 7-percentage point absolute increase in vaccination intentions represents a 30% relative increase, more than twice the effect size found in the same analysis among all participants. Among these historical non-vaccinators, the identifiable victim messages also marginally increased vaccination intentions compared to the general message, *M*_*Identifiable*_ = 32%, 95% CI [30%, 34%] vs. *M*_*General*_ = 29%, 95% CI [27%, 32%], *t* (1195.81) = 1.84, Cohen’s *D* = 0.11, *r* = .05, *p* = .07. Vaccination intention did not differ between the young and old victim conditions among these participants, *p* > .80.

### Country, age, and gender

The effect of message on sympathy, prosocial motivation, and vaccination intentions described above generally held true same in the analyses of covariance (ANOCOVA), where we controlled for age, gender, country, and interaction between country and message (assessing cross-country variations in the effect of message). In the interest of space, detailed analyses on cross-country variations are reported in S2 Supporting Information. Briefly, the effect of message varied across countries only for sympathy, but not on prosocial motives or vaccination intentions. Females and older participants in general showed greater sympathy, prosocial motives, and vaccination intentions, with a few exceptions. One interesting result was that participants living in countries with greater population aging demonstrated a greater gap in sympathy towards the young vs. old victim.

### Mediational analyses on vaccination intentions

We have established that the messages eliciting prosocial motives did increase intentions to vaccinate against flu, especially among those who lacked a recent history of doing so, and more so if the messages involved an identifiable victim. As the central idea in the current study was to test prosocial motives in flu vaccination, we tested whether prosocial motivation was indeed a mediator in these observed effects.

**Fig 4:**
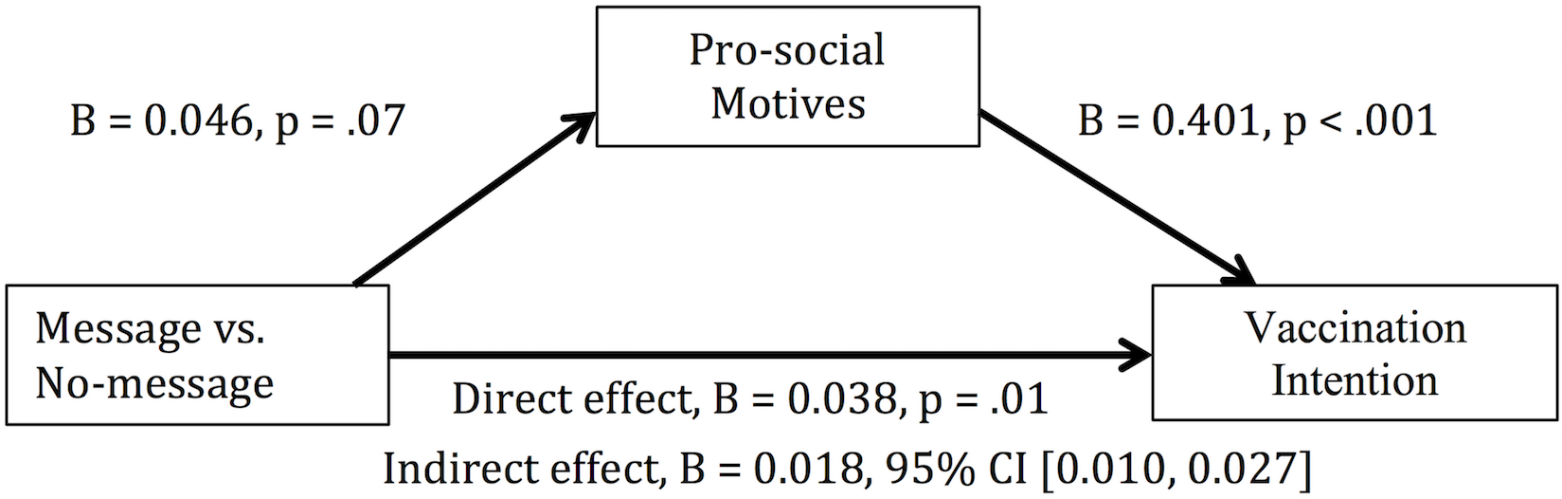
Mediational Model for the effect of message on vaccination intention among all participants, mediated by prosocial motives. This mediation was based on the bootstrapping method for calculating indirect effect developed by Hayes[32]. Participants included anyone with a valid response on vaccination intention (n = 3581). Analysis was performed in SPSS using the PROCESS macro developed by Hayes [32], with 5000 resampling to compute the Bias Corrected boot strapped 95% Confidence Intervals (BCa CI). Total effect of message on vaccination intention was significant, B = 0.056, BCa 95% CI [0.029, 0.083], p = .0001.

First, we performed a mediational analysis including all participants who had a response to the vaccination intention question (n = 3518). The predictor was the contrast between the three message conditions and no message condition, the outcome was vaccination intention, and the mediator was prosocial motive, as assessed by likelihood to donate to an unrelated cause. Based on the PROCESS Macro developed for SPSS by Hayes [32], and using a bias-corrected bootstrapping method with 5000 resampling, the indirect effect was significant, *B* = 0.018, 95% CI [0.010, 0.027] (Fig 4). Thus, compared to the no-message condition, a net increase of 1.8-percentage points in vaccination intentions in the message conditions was attributable to elevated prosociality. Note that the message conditions lead to a 6-percentage increase in vaccination intentions compared to the no message condition. Thus, about one third of this increase was due to the meditational effect of prosocial motives.

We then focused on the 2530 participants who had not vaccinated in the past year, where the effect of message on vaccination intentions was larger. In a similar meditational analysis we found a significant indirect effect of prosocial motives on the desire to be vaccinated (B = 0.020, 95% CI [0.009, 0.030], *κ*^2^ = .031, 95% CI [0.014, 0.046]). This result indicates that among those who did not vaccinate in the past year, a net increase of 2 percentage points in vaccination intentions (out of a total of 7 percentage point increase) was attributable to elevated prosociality.

We did not test sympathy as a potential mediator for the effect of message on vaccination intention, because the sympathy question was directed towards flu victims described in the messages, and therefore, was not measured in the no-message condition.

## Discussion

The results on sympathy confirmed that an identifiable victim in messages elicits increased sympathy, and that people value younger lives more than older lives [29–31]. However, greater sympathy towards younger victim did not translate into greater prosocial motivation. We did find that highlighting the benefit of vaccination to others, as well as using an identifiable victim in the message, can increase motivation to help others, even when the victims highlighted are not the target of helping.

As a focus of this paper, the findings concerning vaccination intentions confirmed the hypothesis that highlighting flu victim increased intentions to vaccinate, especially among people who had not vaccinated the previous year. Among these historical non-vaccinators, reading any of the three messages about victims who suffered from the flu due to others’ failure to vaccinate increased their vaccination intention from 24% to 31%, an absolute increase of 7 percentage points and relative increase of over 30%. Among this group of participants, there was also a marginally significant effect of identifiable victim on the intention to vaccinate. These effects were diluted when the analysis included participants who had vaccinated in the previous year (who were already highly motivated to vaccinate), but the effect of presenting a message (versus not) remained significant: When all participants are considered, reading any of the three messages increased vaccination intentions from 38% to 44%, a 15% relative increase. This might seem to be a small effect, but as a comparison, a recent randomized trial that delivered five weekly text-messages regarding flu vaccination to participants increased vaccination rate from 23% to 27%, an absolute increase of 4 percentage points [33]. Admittedly, the methods of these two studies are very different. However, the fact that their effect sizes fall into similar range highlights prosocial messaging as a potentially effective and viable strategy to promote flu vaccination, especially among people who are historical non-vaccinators.

The mediation analysis showed that prosocial motivation plays a causal role in flu vaccination, and that roughly one third of the effect of message on vaccination intentions occurred due to the elevated prosocial motives. Thus, the current research demonstrated prosociality as a causal factor in flu vaccination, again pointing to the potential of harnessing prosociality in flu vaccination programs or public health campaigns.

What might be the mechanisms through which this type of messaging worked to increase prosociality? As mentioned in the introduction, we designed these messages with the intention to heighten personal responsibility as well as sympathy. Because sympathy was not measured in the no-message condition in the current study, we could not test how sympathy changed in response to our messages versus no message. However, supporting past research on the role of empathetic emotion in prosocial behavior [34], we did find sympathy to be a significant mediator in the effect of identified vs. non-identified victims on prosociality (Fig 2). We did not measure personal responsibility in the current study, as we speculate such a measure may create demand effect, where participants would feel a greater sense of personal responsibility because we asked, and indicate greater likelihood to vaccinate than they otherwise would.

Another possible mechanism in how the messages elicited prosocial motives may be mortality salience, as all our messages mentioned death. Terror Management Theory posits that mortality salience promotes behaviors consistent with cultural standards, and where such standards include prosociality, mortality salience can increase donation [35]. This latter mechanism may be outside of the initial intention of the current study, but may play a role in how the victim-focused messaging leads to prosocial acts. Future research can explore these mechanisms in prosocial messaging about flu vaccination.

The current study is the first to evaluate the use of an identifiable victim as a messaging approach to elicit prosocial behavior directed towards something other than the victim. In contrast, previous research on the identifiable victim effect has focused on prosociality towards the victim only [23–26]. Even though identifiable victim only led to a marginally significant increase in vaccination intentions in the current study, it significantly increased prosocial motives in general, measured by the likelihood to donate towards an unrelated illness. Future studies can explore the domains in which the identifiable victim effect can be harnessed to promote prosocial behavior.

One potential limitation of the study is the lack of an information-only condition that does not elicit prosociality. However, to accurately explain the full effect of vaccination, one must explain herd immunity and thus the benefit of vaccination to others, or importantly, the reduction of risk to others. And this might elicit prosociality. A control condition, therefore, could either provide no information, or provide information on the self-interest benefit of flu vaccination only and ignore herd immunity. Future research could take the latter approach, which would serve as a test for the relative efficacy of self-interest vs. prosociality oriented messages. Given that the goal of the current research was to verify the causal role of prosociality in flu vaccination, not to compare the relative efficacy of prosocial versus self-interest messages, we took the former approach and used the no-message condition as control. And the findings in the meditational analysis described in the end of the results section showed that prosociality indeed played play a causal role, accounting for roughly one third of the messaging effect on vaccination intentions when comparing the three message conditions to the no-message condition.

Another limitation is the use of vaccination intention instead of vaccination behavior as the outcome variable. We did collect follow-up data on flu vaccination behavior in 6 out of the 8 countries, with a response rate of 57%. According to these data, there was no difference in vaccination rate across conditions in these limited data. This lack of effect is not too surprising given the long lag between receiving the message and the actual time in vaccination decisions, and the results may subject to selection bias in responding. Future research should further explore the effectiveness of prosocial messages on behavior at doctors’ offices or pharmacies, where people’s vaccination decisions can be made shortly after exposure to the message.

In summary, the current research provides new insights on the causal role of prosocial motives in vaccination decisions, and suggests that messages intended to elicit prosocial motives may be a useful tool to promote flu vaccination. Importantly, prosocial messaging can be easily incorporated into flu vaccination interventions that have proved to be highly effective, such as provider reminder messages [36] or default vaccination appointments [37] to further motivate vaccination, where the implementation of adding prosocial messages will incur little cost, but may lead to tangible increase in vaccine coverage.

## Supporting Information

S1 Fig Estimated mean sympathy ratings towards flu victim(s) by country in 3 conditions containing message. Means were estimated while controlling for participant age and gender. Error bars: ± 2 Standard Errors.
(TIF)

S2 Fig Estimated mean rating on the likelihood to donate to an unrelated cause by country by message condition. Means were estimated while controlling for participant age and gender. Error bars: ± 2 Standard Errors.
(TIF)

S3 Fig Estimated mean rating on the likelihood to vaccinate this year by country by message condition. Means were estimated while controlling for participant age and gender. Error bars: ± 2 Standard Errors.
(TIF)

S1 Supporting Information Survey Items. Original scenario text and measures used in the study.
(DOCX)

S2 Supporting Information Additional Results. Results from additional analyses.
(DOCX)

S3 Supporting Information Dataset for Open Access. (XLSX)

S4 Supporting Information Codebook for Dataset. (DOCX)
