# Supplementary figures and images for: Stimulating Influenza Vaccination via Prosocial Motives
Source: PLoS One. 2016 Jul 26;11(7):e0159780. doi: 10.1371/journal.pone.0159780 (PMC4961402; doi:10.1371/journal.pone.0159780)

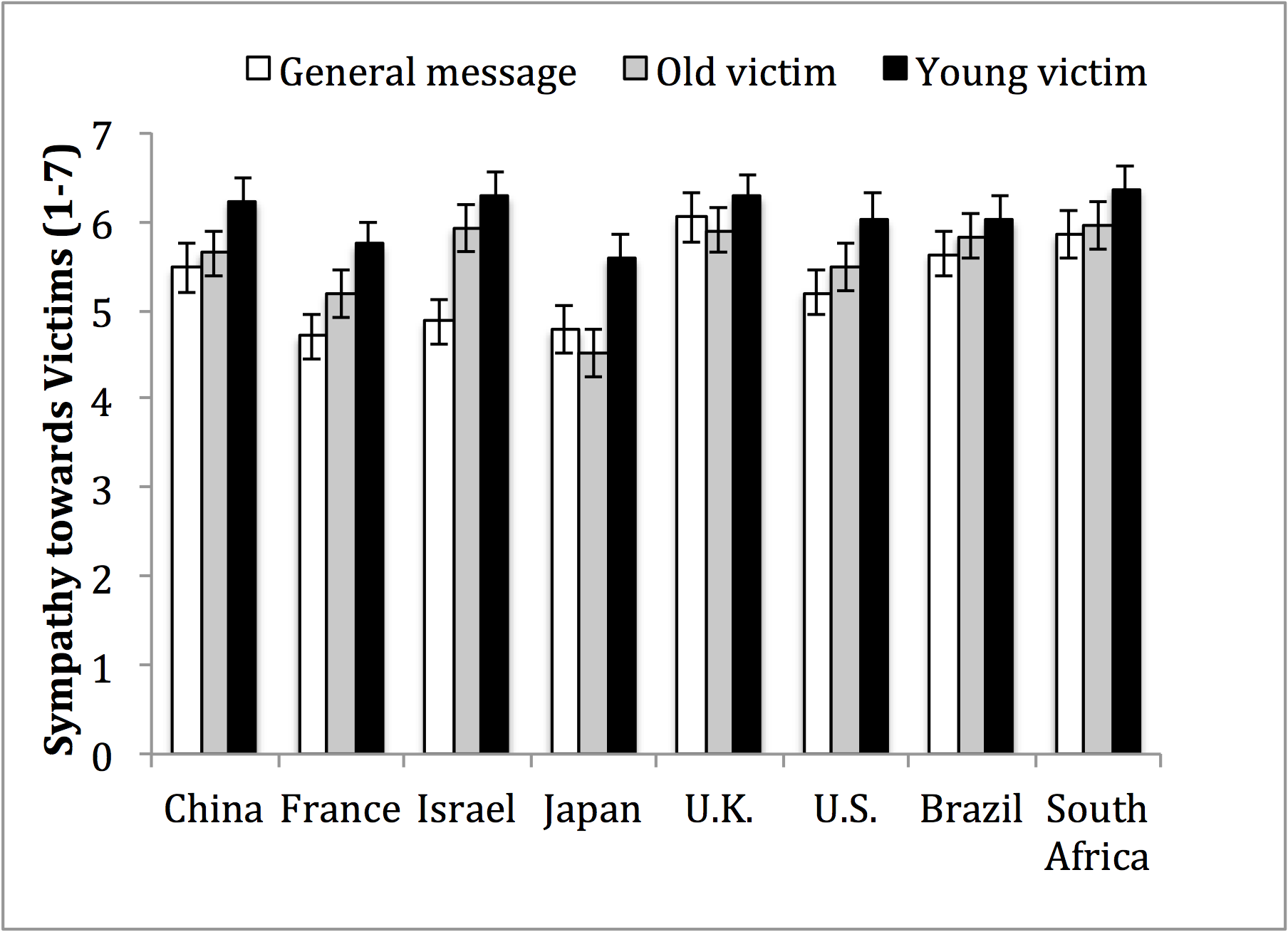

Supplement: S1 Fig — Means were estimated while controlling for participant age and gender. Error bars: ± 2 Standard Errors. (TIF) [file pone.0159780.s001.tif]

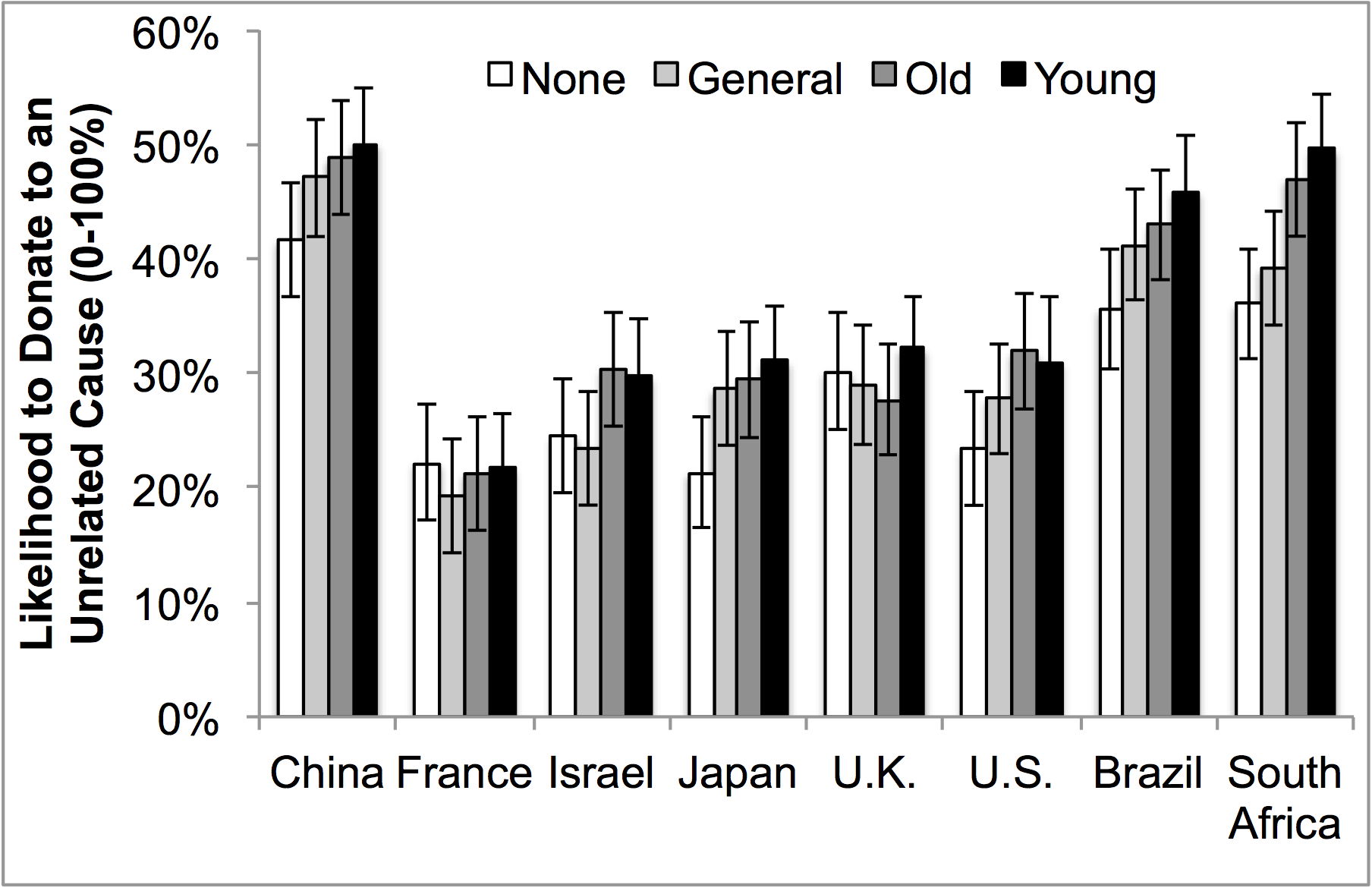

Supplement: S2 Fig — Means were estimated while controlling for participant age and gender. Error bars: ± 2 Standard Errors. (TIF) [file pone.0159780.s002.tif]

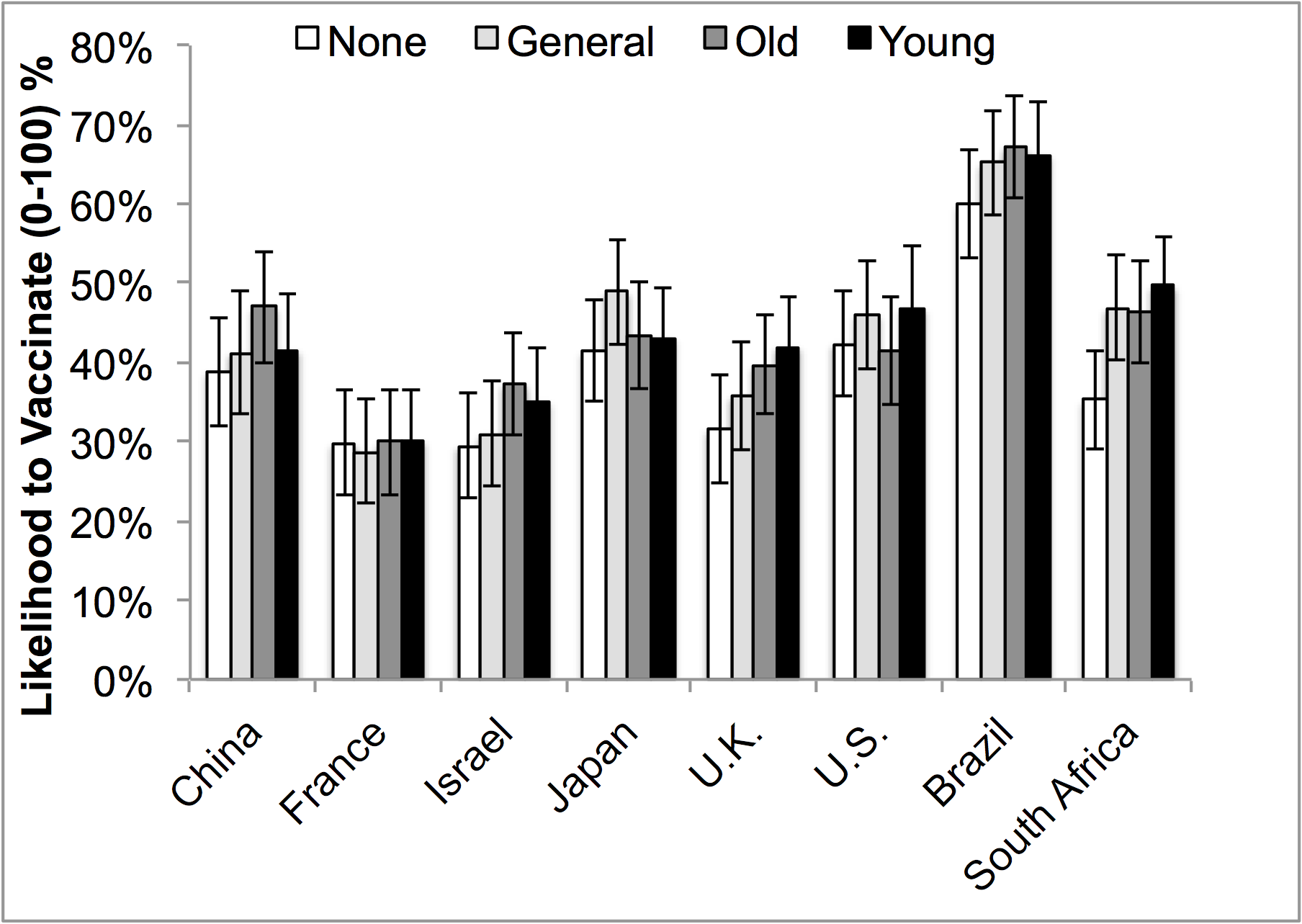

Supplement: S3 Fig — Means were estimated while controlling for participant age and gender. Error bars: ± 2 Standard Errors. (TIF) [file pone.0159780.s003.tif]
