# Supplementary material for: Stimulating Influenza Vaccination via Prosocial Motives
Source: PLoS One. 2016 Jul 26;11(7):e0159780. doi: 10.1371/journal.pone.0159780 (PMC4961402; doi:10.1371/journal.pone.0159780)
Supplement: S1 Supporting Information — Original scenario text and measures used in the study. (DOCX) [file pone.0159780.s004.docx]

**Appendix**

**Messages Used in Study**

***Older Victim Message****:* Walter was 76 years old. He lived alone but was visited frequently by his friends and family who lived close by. Walter was not able to get the flu vaccine last year due to mobility problems. This meant that Walter relied on other people to get vaccinated to reduce the chance that they spread the virus to him. These people included both direct contacts (his friends and family) and indirect contacts (others in contact with friends and family). Last year many people neglected to get the flu vaccine, and Walter became infected. As a result of his infection he acquired pneumonia due to a weakened immune system caused by the flu. He was in a critical condition for several weeks before passing away.

***Younger Victim Message****:* Aiden was 5 months old. He was the youngest of two siblings, and lived with his parents and grandfather. Aiden was not able to get the flu vaccine last year because children under 6 months are not eligible. This meant that Aiden relied on other people to get vaccinated to reduce the chance that they spread the virus to him. These people included both direct contacts (his friends and family) and indirect contacts (others in contact with friends and family). Last year many people neglected to get the flu vaccine, and Aiden became infected. As a result of his infection he acquired pneumonia due to a weakened immune system caused by the flu. He was in a critical condition for several weeks before passing away.

***Non-identified Message:*** Some people find it inconvenient to get the flu vaccine. However, it is important that people get vaccinated, because the flu spreads widely among the population and infects many people. Despite this fact, every year many people do not get vaccinated. In some of these cases, the flu victims encounter additional health problems. For example, they may get pneumonia due to a weakened immune system caused by the flu. When this happens they are often in critical condition for several weeks and may die.

Note: Each message was accompanied with the image of an elderly man, a baby boy, and a male doctor, respectively. We used images of white victims in European and American countries (France, Israel, UK, US, and Brazil), images of Asian victims in China and Japan, and images of black victims in South Africa. All images portrayed male victims.

**Dependent Measures**

***Sympathy****:* Please rate how much sympathy you feel for Walter (Aiden/people who got the flu and developed pneumonia). In other words, how sorry are you that Walter (Aiden) became sick and died (may have died)?

| 1=very little | 7=very much |
| --- | --- |

 sympathy  1 2 3 4 5 6 7

**
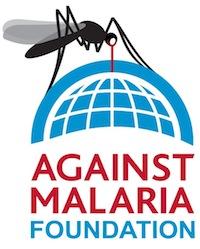
*General Prosocial Motives****:* There is a charitable organization that is called “Against Malaria Foundation”. This charity provides insecticidal nets (a net that offers protection against insects and thus against the diseases they may carry) to communities in need.

How likely are you to donate to this charity?

**NOTE:**You will NOT be contacted by this charity

| 0% | 10% | 20% | 30% | 40% | 50% | 60% | 70% | 80% | 90% | 100% |
| --- | --- | --- | --- | --- | --- | --- | --- | --- | --- | --- |

***Vaccination Intentions****:* How likely are you to get the flu vaccine this coming flu season? Try to be objective. Consider not only the potential benefits of getting the flu vaccine, but also any reservations you may have, and the practicalities involved, such as making the appointment and going to the clinic.

| 0% | 10% | 20% | 30% | 40% | 50% | 60% | 70% | 80% | 90% | 100% |
| --- | --- | --- | --- | --- | --- | --- | --- | --- | --- | --- |
