# Supplementary material for: Stimulating Influenza Vaccination via Prosocial Motives
Source: PLoS One. 2016 Jul 26;11(7):e0159780. doi: 10.1371/journal.pone.0159780 (PMC4961402; doi:10.1371/journal.pone.0159780)
Supplement: S2 Supporting Information — Results from additional analyses. (DOCX) [file pone.0159780.s005.docx]

**S1-Supporting Materials**

**Results in cross-country variations in the effect of message**

To account for the cross-country variations, including the variation on age and gender across countries, we performed analyses on our key dependent variables with participant country, age, and gender controlled for. In addition, we tested the interaction between country and message condition to see whether the effect of message varied systematically in different countries.

**Sympathy**

Because participants from different countries vary on age and gender composition, to examine cross-country differences in the effect of message on sympathy, we conducted an Analysis of Covariance (ANCOVA) analysis on sympathy with condition and country as the independent variables, and participant age and gender as covariates. The analysis included all participants in the three message conditions, where sympathy was measured (n = 2968). Message and country had a significant interaction *F* (14, 2942) = 3.58, *p* < .001 (albeit Levene’s test showed that there was significant heterogeneity of variance across groups), and thus, participants from different countries did respond differently to the different messages.

We next examined whether participants from different countries respond differently to messages with identified vs. non-identified victims. As S1 Fig illustrates, in most countries, participants were more sympathetic in the two identified victim conditions than in the general condition. Indeed, the contrast between the general message vs. the two identified victim conditions was significant or marginally significant (p < .10) in all countries except in Japan (contrast = 0.53, 95% CI [-0.10, 1.17], p = .10) and U.K. (contrast = 0.09, 95% CI [-0.55, 0.74], p = .77). There is no obvious pattern in the results that conforms to the distinction between collectivist and individualist countries, despite recent findings that the identifiable victim effect may only exist in individualist countries (27)

**S1 Fig**. **Estimated mean sympathy ratings towards flu victim(s) by country in 3 conditions containing message.** Means were estimated while controlling for participant age and gender. Error bars: ± 2 Standard Errors

The current study allows us to investigate cross-national variations in age attitudes. There has been much research showing that Americans value the lives of younger people more than those of older people (22-24). And, despite common beliefs, a recent meta-analysis found that eastern countries hold more negative views of the elders compared to western countries, and this difference has widened with time (North, 2013). To test whether these findings held true in our data, we analyzed how participants from different countries respond to the young victim message vs. the old victim message. As Fig S1 illustrates, in most countries, participants were more sympathetic towards the young victim than the old victim, except in Brazil, where this contrast was the smallest and not significant, Mean Difference _Young - Old_ = 0.18, 95% CI [-0.19, 0.55], *p* = .34. On the other hand, this effect was largest for Japan, Mean Difference _Young - Old_ = 1.08, 95% CI [0.71, 1.44], *p* < .001. But China (Mean Difference _Young - Old_ = 0.58, 95% CI [0.23, 0.94]), another eastern country, showed a difference that was similar in size to U.S. (Mean Difference _Young - Old_ = 0.53, 95% CI [0.14, 0.93]) and France (Mean Difference _Young - Old_ = 0.56, 95% CI [0.19, 0.92]). Therefore, the pattern of sympathy results did not fit a clear pattern where eastern and western countries differ in their differential response to young vs. old victims [38].

However, the largest gap in sympathy between young and old victims occurred in Japan, where the problem of population aging is well known (Median Age = 46.1, retrieved from Central Intelligence Agency, or CIA), and the smallest gap occurred in Brazil, where the population is much younger (Median age = 29.9). This difference in sympathy may reflect negative attitudes towards older people in the population (compared to young victim) due to the burden of population aging. To test this population aging hypothesis, we performed a regression analysis, using condition contrasts (Contrast 1: Young vs. Old victim; Contrast 2: Identified victim conditions vs. general victim condition), individual characteristics (participant’s age and gender) and country age ( median population age of the country), as well as the interaction between contrast 1 and each of the three individual and country characteristics. As listed in Table S1, condition contract 1 had a significant effect on sympathy, B = 0.50, SE B = 0.07, partial *η^2^* = 0.02, p < .01, indicating that on average, sympathy increased 0.50 points on a 7-point scale for the young victim compared to old victim. Condition contract 2 also had a significant positive coefficient, B = 0.51, SE B = 0.06, partial *η^2^* = 0.02, p < .01, indicating that there is a significant identifiable victim effect, where identifiable victim (in the young victim and old victim conditions) elicited greater sympathy than the general victim condition (by 0.51 points). These results are consistent with the analyses on identifiable victim effect presented earlier, as well as those described in the main text.

**S1 Table. Regression of sympathy rating on message condition, participant characteristics and country median age.**

|  | *B* | *SE B* | *β* | *t* | *p* | *sp η^2^** |
| --- | --- | --- | --- | --- | --- | --- |
| Contrast 1  (Young vs. Old victims) | 0.50 | 0.07 | 0.13 | 7.38 | < .01 | 0.02 |
| Contrast 2  (Identified vs. generic victims) | 0.51 | 0.06 | 0.15 | 8.67 | < .01 | .02 |
| Participant's Age (Age_p) | 0.01 | 0.002 | 0.12 | 6.59 | < .01 | .01 |
| Country Median Age (Age_c) | -0.04 | 0.004 | -0.16 | -9.07 | < .01 | .03 |
| Participant's gender | 0.47 | 0.06 | 0.15 | 8.47 | < .01 | .02 |
| Age_p × Age_c | 0.00 | 0.00 | 0.02 | 1.34 | .18 | < .001 |
| Contrast 1 × Age_p | -0.01 | 0.01 | -0.02 | -1.22 | .22 | < .001 |
| Contrast 1 × Age_c | 0.03 | 0.01 | 0.05 | 2.81 | .01 | .003 |
| Contrast 1 × gender | 0.21 | 0.14 | 0.03 | 1.57 | .12 | < .001 |
| Contrast 1 × Age_p × Age_c | 0.00 | 0.00 | 0.01 | 0.57 | .57 | < .001 |

All continuous predictors were centered on the mean, and all contrasts were structured with 0.5 assigned to the group mentioned first, and -0.5 assigned to the group mentioned last). Age_p: Participant age; Age_c: Country median age. Participant’s gender: -0.5=male, 0.5=female.

**sp η^2^* : semi-partial *η^2^*.

Participant’s own age and gender also affected mean sympathy rating towards the victim, with older participants and females more sympathetic in general, B = 0.01, SE B = 0.002, partial *η^2^* = 0.01, p < .01, and B = 0.47, SE B = 0.06, partial *η^2^* = 0.02, p < .01, respectively. Participants from countries with greater population aging were less sympathetic in general, B = -0.04, SE B = 0.004, partial *η^2^* = 0.03, p < .01. Participants’ own age did not interact with condition contrast 1, that is, young and old participants alike showed the gap in sympathy towards young and old victims, *p* = .22. Of critical interest, however, population median age interacted with condition, B = 0.03, SE B = 0.01, partial *η^2^* = .003, *p* = .01, where participants in countries with an older population showed greater gap in their sympathy towards the young and old victims. This result supports our hypothesis that greater population aging intensifies the negative responses people have towards old victims compared to young victims.

To summarize, the cross-national sympathy data showed that in general, people demonstrated greater sympathy in messages using an identifiable victim vs. a general message with unidentified victims, and greater sympathy for young victim compared to old victim. The identifiable victim effect was weakest in Japan and U.K, with no other apparent pattern. The preference for young victim was the greatest in Japan and weakest in Brazil, and the pattern was associated with population aging, where countries with greater population aging showed greater favoritism towards the young vs. old victims.

**Prosocial Motives**

We conducted an ANCOVA analysis on vaccination intentions as we did on sympathy. The ANCOVA (albeit with significant heterogeneity of variance) included message condition and country as the independent variables, and participant age and gender as covariates. The analysis included all participants (n = 3952). See Fig S2 for mean prosocial motives by condition by country. Consistent with the analysis in the main text, message had a significant main effect, *F* (3, 3918) = 12.70, *p* < .001. Country also had a significant main effect, *F* (3, 3918) = 12.70, *p* < .001, suggesting variation on baseline willingness to donate to an irrelevant cause, *F* (7, 3918) = 54.25, *p* < .001. However, message and country did not have a significant interaction in predicting prosocial motives, indicating that countries did not have substantial variation in the effect of message on prosocial motives. Age did not correlate with prosocial motives, but gender did, *F* (1, 3918) = 8.20, *p* = .004, with females showing greater prosocial motives in general than males.

**S2 Fig**. **Estimated mean rating on the likelihood to donate to an unrelated cause by country by message condition.** Means were estimated while controlling for participant age and gender. Error bars: ± 2 Standard Errors

**Vaccination Intentions**

We conducted an ANCOVA analysis on vaccination intentions as we did on sympathy and prosocial motivation. As before, the ANCOVA included message condition and country as the independent variables, and participant age and gender as covariates. The analysis included all participants with a response on the outcome measure (n = 3952). See Fig S3 for mean vaccination intentions by condition by country. Consistent with analysis in the main text, the analysis (albeit with significant heterogeneity of variance)message had a significant main effect, *F* (3, 3484) = 5.17, *p* = .001. Country also had a significant main effect, *F* (7, 3484) = 40.25, *p* < .001, suggesting variation on baseline intentions to vaccinate. However, message and country did not have a significant interaction, *F* (21, 3484) = 0.78, *p* = .75, indicating that effect of message on vaccination intentions did not vary significantly across countries. Age and gender both predicted vaccination intentions, so that older people were more likely to vaccinate, *F* (1, 3484) = 33.41, *p* < .001, and females were more likely to vaccinate *F* (1, 3484) = 6.29, *p* = .01. These conclusions held true when the analysis only included those who had not vaccinated in the prior year, except the age and gender effects became marginally significant.

**S3 Fig**. **Estimated mean rating on the likelihood to vaccinate this year by country by message condition.** Means were estimated while controlling for participant age and gender. Error bars: ± 2 Standard Errors

**Supplemental References**

38. North MS, Fiske ST. Modern attitudes toward older adults in the aging world: A cross-cultural meta-analysis. Psychological Bulletin. 2015;141(5):993-1021.
