# Supplementary material for: Stimulating Influenza Vaccination via Prosocial Motives
Source: PLoS One. 2016 Jul 26;11(7):e0159780. doi: 10.1371/journal.pone.0159780 (PMC4961402; doi:10.1371/journal.pone.0159780)
Supplement: S4 Supporting Information — (DOCX) [file pone.0159780.s007.docx]

**Codebook for Dataset**

| **country** | | | | |  |
| --- | --- | --- | --- | --- | --- |
|  | | Value | Count | Percent | |
| Standard Attributes | Position | 1 |  |  | |
|  | Label | <none> |  |  | |
|  | Type | String |  |  | |
|  | Format | A6 |  |  | |
|  | Measurement | Nominal |  |  | |
|  | Role | Input |  |  | |
| Valid Values | Brazil |  | 490 | 12.4% | |
|  | Chi |  | 499 | 12.6% | |
|  | France |  | 497 | 12.6% | |
|  | Israel |  | 500 | 12.7% | |
|  | Japan |  | 501 | 12.7% | |
|  | SA |  | 496 | 12.6% | |
|  | UK |  | 496 | 12.6% | |
|  | US |  | 473 | 12.0% | |

| **gender** | | | | | | | |  |  |
| --- | --- | --- | --- | --- | --- | --- | --- | --- | --- |
|  | | Value | | Count | | Percent | | |  |
| Standard Attributes | Position | | 2 | |  | |  | | |
|  | Label | | <none> | |  | |  | | |
|  | Type | | String | |  | |  | | |
|  | Format | | A6 | |  | |  | | |
|  | Measurement | | Nominal | |  | |  | | |
|  | Role | | Input | |  | |  | | |
| Valid Values | female | |  | | 2063 | | 52.2% | | |
|  | male | |  | | 1889 | | 47.8% | | |

| **age** | | | |
| --- | --- | --- | --- |
|  | | Value |  |
| Standard Attributes | Position | 3 |  |
|  | Label | <none> |  |
|  | Type | Numeric |  |
|  | Format | F11 |  |
|  | Measurement | Scale |  |
|  | Role | Input |  |
| N | Valid | 3952 |  |
|  | Missing | 0 |  |
| Central Tendency and Dispersion | Mean | 38.24 |  |
|  | Standard Deviation | 12.827 |  |
|  | Percentile 25 | 28.00 |  |
|  | Percentile 50 | 35.00 |  |
|  | Percentile 75 | 46.00 |  |

| **Vlast** | | | | |
| --- | --- | --- | --- | --- |
|  | | Value | Count | Percent |
| Standard Attributes | Position | 4 |  |  |
|  | Label | **Flu vaccination status in the previous year** |  |  |
|  | Type | Numeric |  |  |
|  | Format | F11 |  |  |
|  | Measurement | Nominal |  |  |
|  | Role | Input |  |  |
| **Valid Values** | **0** | **Did not vaccinate last year** | 2599 | 65.8% |
|  | **1** | **Vaccinated last year** | 1252 | 31.7% |
| Missing Values | System |  | 101 | 2.6% |

| **donate** | | | | |
| --- | --- | --- | --- | --- |
|  | | Value | Count | Percent |
| Standard Attributes | Position | 5 |  |  |
|  | Label | **Likelihood to donate to an unrelated cause (Against Malaria Foundation), from 0-100 in percentage, with 10% intervals** |  |  |
|  | Type | Numeric |  |  |
|  | Format | F11 |  |  |
|  | Measurement | Nominal |  |  |
|  | Role | Input |  |  |
| Valid Values | 0 |  | 971 | 24.6% |
|  | 10 |  | 517 | 13.1% |
|  | 20 |  | 342 | 8.7% |
|  | 30 |  | 338 | 8.6% |
|  | 40 |  | 224 | 5.7% |
|  | 50 |  | 731 | 18.5% |
|  | 60 |  | 208 | 5.3% |
|  | 70 |  | 216 | 5.5% |
|  | 80 |  | 191 | 4.8% |
|  | 90 |  | 87 | 2.2% |
|  | 100 |  | 127 | 3.2% |

| **dote.amt** | | | | |
| --- | --- | --- | --- | --- |
|  |  | Value | Count | Percent |
| Standard Attributes | Position | 6 |  |  |
|  | **Label** | **Amount (in local currency) willing to donate to the Against Malaria Foundation** | | |

| **Vnext.already** | | | | | | | |  |
| --- | --- | --- | --- | --- | --- | --- | --- | --- |
|  | | | Value | | Count | | Percent | |
| Standard Attributes | Position | 7 | |  | |  | |  |
|  | Label | **Already vaccinated for the upcoming flu season, or not** | |  | |  | |  |
|  | Type | Numeric | |  | |  | |  |
|  | Format | F11 | |  | |  | |  |
|  | Measurement | Nominal | |  | |  | |  |
|  | Role | Input | |  | |  | |  |
| **Valid Values** | **0** | **Have not vaccinated for the upcoming flu season** | | 3491 | | 88.3% | |  |
|  | **1** | **Already vaccinated for the upcoming flu season** | | 372 | | 9.4% | |  |
| Missing Values | System |  | | 89 | | 2.3% | |  |

| **Vnext** | | | | |
| --- | --- | --- | --- | --- |
|  | | Value | Count | Percent |
| Standard Attributes | Position | 8 |  |  |
|  | Label | **Liklihood to vaccinate in the upcoming flu season (if not already vaccinated), from 0-100 in percentage, with 10% interverls** |  |  |
|  | Type | Numeric |  |  |
|  | Format | F11 |  |  |
|  | Measurement | Nominal |  |  |
|  | Role | Input |  |  |
| Valid Values | 0 |  | 822 | 20.8% |
|  | 10 |  | 400 | 10.1% |
|  | 20 |  | 240 | 6.1% |
|  | 30 |  | 231 | 5.8% |
|  | 40 |  | 148 | 3.7% |
|  | 50 |  | 455 | 11.5% |
|  | 60 |  | 165 | 4.2% |
|  | 70 |  | 168 | 4.3% |
|  | 80 |  | 211 | 5.3% |
|  | 90 |  | 158 | 4.0% |
|  | 100 |  | 520 | 13.2% |
| Missing Values | System |  | 434 | 11.0% |

| **message** | | | | |
| --- | --- | --- | --- | --- |
|  | | Value | Count | Percent |
| Standard Attributes | Position | 9 |  |  |
|  | Label | **Message condition** |  |  |
|  | Type | String |  |  |
|  | Format | A5 |  |  |
|  | Measurement | Nominal |  |  |
|  | Role | Input |  |  |
| Valid Values | **doc** | **General flu message, with a doctor's image** | 983 | 24.9% |
|  | **none** | **No message** | 984 | 24.9% |
|  | **old** | **Old victim message, with an old victim's image** | 989 | 25.0% |
|  | **young** | **Young victim message, with a young victim's image** | 996 | 25.2% |

| **sympathy** | | | | |
| --- | --- | --- | --- | --- |
|  | | Value | Count | Percent |
| Standard Attributes | Position | 10 |  |  |
|  | Label | **Rated sympathy towards the flu victim(s), 1(very little)-7(very much)** |  |  |
|  | Type | Numeric |  |  |
|  | Format | F11 |  |  |
|  | Measurement | Nominal |  |  |
|  | Role | Input |  |  |
| Valid Values | 0 |  | 1 | 0.0% |
|  | 1 |  | 74 | 1.9% |
|  | 2 |  | 104 | 2.6% |
|  | 3 |  | 135 | 3.4% |
|  | 4 |  | 318 | 8.0% |
|  | 5 |  | 470 | 11.9% |
|  | 6 |  | 615 | 15.6% |
|  | 7 |  | 1251 | 31.7% |
| Missing Values | System |  | 984 | 24.9% |

| **V_followup.YN** | | | | |
| --- | --- | --- | --- | --- |
|  | | Value | Count | Percent |
| Standard Attributes | Position | 11 |  |  |
|  | Label | **From follow-up survey, whether subjects indeed received vaccination in the current flu season** |  |  |
|  | Type | Numeric |  |  |
|  | Format | F11 |  |  |
|  | Measurement | Nominal |  |  |
|  | Role | Input |  |  |
| Valid Values | **0** | **No** | 894 | 22.6% |
|  | **1** | **Yes** | 598 | 15.1% |
| Missing Values | System |  | 2460 | 62.2% |
